# Automatic Detection and Segmentation of Thrombi in Abdominal Aortic Aneurysms Using a Mask Region-Based Convolutional Neural Network with Optimized Loss Functions

**DOI:** 10.3390/s22103643

**Published:** 2022-05-10

**Authors:** Byunghoon Hwang, Jihu Kim, Sungmin Lee, Eunyoung Kim, Jeongho Kim, Younhyun Jung, Hyoseok Hwang

**Affiliations:** 1 Department of Software Convergence, Kyung Hee University, Yongin 17104, Korea; david.hwang@khu.ac.kr; 2 Department of Software, Gachon University, Seongnam 13120, Korea; paransky9577@gachon.ac.kr (J.K.); yugioh1118@gachon.ac.kr (S.L.); 3 Department of Radiology, Gachon University Gil Medical Center, Incheon 21565, Korea; oneshot0229@gilhospital.com (E.K.); ho7ok7@gilhospital.com (J.K.)

**Keywords:** abdominal aortic aneurysm (AAA), optimized loss function, CTA images, thrombus detection and segmentation, Mask R-CNN

## Abstract

The detection and segmentation of thrombi are essential for monitoring the disease progression of abdominal aortic aneurysms (AAAs) and for patient care and management. As they have inherent capabilities to learn complex features, deep convolutional neural networks (CNNs) have been recently introduced to improve thrombus detection and segmentation. However, investigations into the use of CNN methods is in the early stages and most of the existing methods are heavily concerned with the segmentation of thrombi, which only works after they have been detected. In this work, we propose a fully automated method for the whole process of the detection and segmentation of thrombi, which is based on a well-established mask region-based convolutional neural network (Mask R-CNN) framework that we improve with optimized loss functions. The combined use of complete intersection over union (CIoU) and smooth L1 loss was designed for accurate thrombus detection and then thrombus segmentation was improved with a modified focal loss. We evaluated our method against 60 clinically approved patient studies (i.e., computed tomography angiography (CTA) image volume data) by conducting 4-fold cross-validation. The results of comparisons to multiple other state-of-the-art methods suggested the superior performance of our method, which achieved the highest F1 score for thrombus detection (0.9197) and outperformed most metrics for thrombus segmentation.

## 1. Introduction

An abdominal aortic aneurysm (AAA) is an expansion of the abdominal aorta of more than 50% of its normal diameter, which is caused by weakened arterial walls [1]. AAAs are the 14th most common cause of mortality in the United States and are a significant public health issue [2]. After the age of 50, the incidence of AAAs increases steadily and is 2–3 times more likely to occur in males [3,4]. In the majority of cases, AAAs dilate continually without causing any symptoms. However, when blood leaks between the arterial walls or a portion of the artery ruptures, the mortality rate rise to more than 80% [5,6]. To prevent possible leaks or ruptures, one available treatment relies on a surgical approach that involves open aneurysm repair (OAR), while another is a minimally invasive technique that is known as endovascular aneurysm repair (EVAR) [7]. Open aneurysm repair is a traditional surgical treatment that removes the aneurysm completely through open surgery and replaces it with a synthetic vessel. Endovascular aneurysm repair does not require open surgery and instead, a stent graft is inserted through the vessel to prevent rupture by allowing the aneurysm to flow into the stent graft. The treatment method is only chosen after carefully considering anatomic characteristics and the age, gender, concomitant diseases, and mortality of the patient.

A synthetic stent graft is placed inside the aorta after surgical treatment and damage or fatigue in the graft material can induce leakage, graft migration or graft twisting, which can lead to rupture or occlusion. As a result, depending on the diameter and state of the thrombus, the patient should be checked every 3 to 12 months [8]. Computed tomography angiography (CTA) is the currently preferred imaging method for the diagnosis of changes in thrombus volume or ruptures and trained radiologists manually perform to detect aneurysms and measure the thrombus diameter for each slice that is obtained through CTA [9,10]. However, CTA images are challenging for the following reasons:

- As thrombi have irregular morphologies, precise segmentation is essential;
- Similar intensity values make distinguishing a thrombus from surrounding tissues challenging;
- Due to the thrombus being obscured by the metal stent graft, it becomes difficult to detect and segment;
- Manual labeling takes a long time, even for expert radiologists, and data are limited.

**Figure 1:**
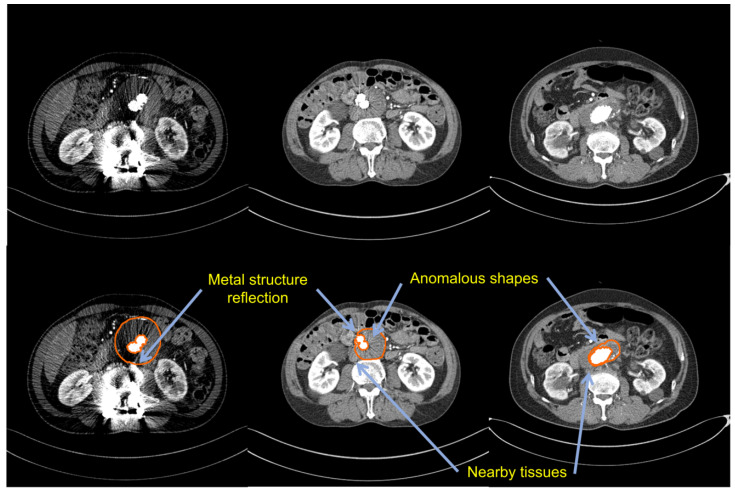
A sample training image from our dataset: the first row shows the original images; the second row describes the challenging features of CTA images.

Figure 1 shows the characteristics that are mentioned above. As a result, graph cuts, level sets, and deformable models, which are traditional methods that use intensity information with shape constructions, are difficult to use for the accurate segmentation of thrombi that have similar intensities to surrounding tissues [11,12,13,14,15,16]. As most of these methods rely highly on several parameter adjustments, they have impacts on clinical applications. Trained radiologists can also only detect thrombi with a 65% degree of accuracy and it is time-consuming manual work [17].

Deep convolutional neural networks (DCNNs) have become famous for their remarkable success in computer visualization processes, such as image classification, object detection, and image segmentation [18,19,20]. In addition, the deep learning-based method for thrombus detection and segmentation has also achieved high levels of performance. However, most of the methods that use deep learning focus on thrombus segmentation rather than thrombus detection. Few studies have obtained segmentation results following thrombus detection, which is far more clinically relevant than segmentation alone.

In this paper, we present a novel method for thrombus detection and segmentation that is based on an improved Mask R-CNN. This method could accurately detect thrombi in CTA image slices and segment thrombi of irregular shapes. Furthermore, the proposed method improved detection performance by introducing a bounding box regression loss function that combined smooth $L_{1}$ loss [21] and complete intersection over union (CIoU) [22]. Weighted binary focal loss as a mask loss function improved segmentation performance by decreasing mis-segmentation. As a result, the proposed method could help radiologists in terms of overall diagnosis by performing high-accuracy thrombus detection and segmentation while also reducing analysis time.

The rest of this paper is organized as follows. In Section 2, we review the other methods that are related to thrombus detection and segmentation. In Section 3, we describe the proposed method in detail. Next, in Section 4, we describe our dataset, environment settings, evaluation method, and the evaluation results. Finally, our conclusions and directions for future work are explained in Section 5.

## 2. Related Works

In order to monitor the growth rates of thrombi, they need to be diagnosed using various imaging methods and tests, both pre-operative and post-operative. A chest X-ray is one of the basic tests for diagnosing asymptomatic aortic aneurysms and is used for initial diagnosis. However, these X-rays are not suitable for accurate thrombus detection or follow-up. Magnetic resonance imaging (MRI) has the advantage of being able to determine the condition of the aorta without using a contrast, but the examination takes a long time and is difficult to perform when a patient’s condition is deteriorating rapidly [23,24,25,26,27]. On the other hand, CTA enables rapid examination, has a high resolution, and can obtain three-dimensional images of thrombi and other important adjacent vascular structures, all of which leads to breakthrough progress in AAA diagnosis.

Thrombus detection and segmentation cause significant challenges due to the size and shape variability of thrombi and in the differentiation of thrombi from surrounding tissues. Several semi-automatic and fully automatic methods have been proposed to solve these problems. As a semi-automatic method, Bruijne et al. [28] presented an active shape model formulation in which landmarks are defined by comparing nearby slices instead of training data. The first slice is manually segmented and then the slice outline of the entire aneurysm is automatically detected. Macia et al. [29] used radial functions that were constrained by a priori knowledge and spatial coherency as a new model-based approach to the semi-automatic segmentation of both the lumen and thrombus of an AAA. User interaction is minimized by defining the two seed points that are contained within the lumen and defining the range of slices of interest. Joldes et al. [30] used a finite element analysis that was based on user-entered parameters, such as the thickness of the AAA wall, the inclusion of the thrombus, and geometry meshing. They created a software system called BioPARR, in which the entire analysis is automatically performed except for the semi-automatic segmentation of the AAA. Lalys et al. [14] proposed a method to first detect the centerlines in order to obtain the initial lumen segmentation, which requires minimal user interaction. The thrombus and lumen are then separated from the surrounding structures using gradient information during the pre-processing step. The final segmentation is performed using a deformable model.

Regarding fully automatic methods, Zheng et al. [31] trained the UNet using only a small dataset and obtained successful segmentation results. They found that overfitting could be avoided through data augmentation with gray value variation and translation. Hong et al. [32] proposed a fully automatic method for the detection and segmentation of aneurysm using a deep belief network-based approach. Wang et al. [33] proposed a novel network that fuses the high-level part of the CT and MRI image networks together based on the UNet architecture. They demonstrated that their fusion model increases the ability to learn the shared representations of multi-modality images. Lopez-Linares et al. [34] presented a two-dimensional automatic method that uses different networks for detection and segmentation. DetectNet is only used for the detection of AAA thrombi, while segmentation is performed using a modified holistically nested edge detection (HED) network. Even more relevant to this paper, Lu et al. [35] presented a three-dimensional algorithm for AAA segmentation for the first time. The detection and segmentation are performed by applying ellipse fitting that is based on a variant of the 3D UNet architecture.

Furthermore, various other methods have also recently achieved state-of-the-art medical image segmentation. The appropriate loss function for each method is combined with the ensemble and transformer methods to demonstrate good overall performances. Nanni et al. [36] proposed encoder–decoder ensemble classifiers that can be used for semantic segmentation and introduced a novel loss function that results from the combination of Dice loss and a structural similarity index (SSIM). Dong et al. [37] presented a pyramid vision transformer backbone as an encoder for the extraction of robust features that has three tight components: a cascaded fusion module (CFM), camouflage identification module (CIM), and similarity aggregation module (SAM). The sum of the IoU and weighted binary cross-entropy loss is used as the loss function.

In this paper, we present a novel loss function from the Mask R-CNN framework. We also present an efficient loss function that improved the performance of thrombus detection and segmentation by considering various factors, such as the aspect ratio and overlap area of the predicted bounding box, when used in a region-based convolutional neural network method that consisted of two stages. Moreover, we trained the architecture using 2D images by slicing a limited 3D CTA dataset.

## 3. Materials and Methods

### 3.1. Structure of Mask R-CNN

Our ultimate goal was to only precisely detect and process the thrombus out of a wide range of CTA datasets, which were located from the heart to the pelvis. Our method was based on Mask R-CNN, which is one of the most popular frameworks for detection and segmentation and takes into consideration certain difficulties that can occur in CTA images, such as irregular shapes, stent graft occlusions, and the differentiation of thrombi from adjacent tissues [38].

**Figure 2:**
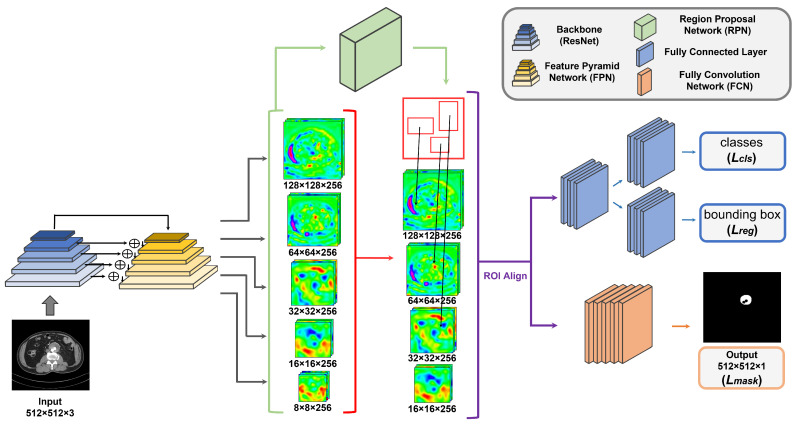
An overview of the workflow for thrombus detection and segmentation using the Mask R-CNN framework.

The Mask R-CNN consisted of several sub-modules, including the backbone network, feature pyramid network (FPN) [39], region proposal network (RPN), classifier network, and mask generation network, as shown in Figure 2. The backbone used ResNet50 [40] to extract the abstract features of CTA images of thrombi through convolution operation. At this time, feature maps of various sizes were obtained and FPN could then obtain five feature maps after gradually merging all of the initial feature maps. During the RPN stage, three different ratios of anchors were used to create a region of interest (ROI), which was the area where the foreground was likely to be. The ROI preserved the spatial location through RoIAlign in order to minimize the differences in misalignment between the extracted features and the fixed grid sizes. Next, the ROI that was generated from the RPN went through the RoIAlign layer to obtain a refined feature map, which was then forwarded to each of the two sub-modules. One sub-module was a classifier that distinguished between the background and class of each ROI and regression, which predicted the bounding box using the ground truth (GT). The other sub-module was a fully convolutional network (FCN) [41] and was used to predict the segmentation masks for each class. Formally, Mask R-CNN was used as a multi-task loss function for training that consisted of the sum of classification loss $L_{cls}$, bounding box regression loss $L_{reg}$, and segmentation mask loss $L_{mask}$, which was defined as:

(1) $$L=L_{cls}+L_{reg}+L_{mask}.$$

The cross-entropy function was used to compute the classification loss and the smooth $L_{1}$ loss was used to calculate the bounding box regression loss, which was the same as the loss function in Faster R-CNN [42]. $L_{mask}$ was defined as the average binary cross-entropy, which was defined as:

(2) $$L_{mask}=-\frac{1}{m^{2}}\sum_{1\leq i,j\leq m}\left[y_{ij}\log{\hat{y}}_{ij}^{k}+\left(1-y_{ij}\right)\log\left(1-{\hat{y}}_{ij}^{k}\right)\right],$$

where $y_{ij}$ represents the ground truth value for the area of m×m and ${\hat{y}}_{ij}^{k}$ is the prediction value for the *k*-th class in the mask. The mask prediction did not have to compete between the *k*-th class, so it was not affected by each class result.

### 3.2. Improvement of Loss Function

Specific learning objectives need to be considered after the network topology has been determined. As an objective function of the optimization problem, machine learning generally uses a function that is known as a loss function. This is important in deep learning because it determines how to update the network parameters based on the purpose. Setting a reasonable loss function is critical and changing the loss function to only suit each specific purpose can improve the overall performance of the network. As shown in Equation (1), the Mask R-CNN in our study consisted of three loss functions. Among the three loss functions, $L_{reg}$ and $L_{mask}$ were redesigned to take into consideration the heterogeneity of the thrombus morphology and data characteristics. The bounding box regression loss was modified to accurately identify a thrombus from the surrounding tissue. The segmentation loss was changed to reduce the risk of the mis-segmentation of the thrombus. The classification loss was unchanged because the dataset only contained two classes: thrombus and background.

The bounding box regression loss function of the basic Mask R-CNN model is used as the smooth $L_{1}$ loss. Compared to the widely used $L_{2}$ loss, the smooth $L_{1}$ loss reduces sensitivity to outliers and is often used as the bounding box loss for two-stage methods, such as Faster R-CNN and Mask R-CNN. The smooth $L_{1}$ loss is defined as follows:

(3) $$smooth_{\ell_{1}}\left(x\right)=\left\{\begin{array}{cc} 0.5x^{2}, & \;\mathrm{if}\;\left|x\right|<1\\ |x|-0.5, & \;\mathrm{otherwise}.\; \end{array}\right.$$

However, the value of smooth $L_{1}$ loss calculates all four bounding box points as independent variables, so the correlation between each point disappears. It also has a high value when the coordinate value is large because it is not normalized.

The IoU-based loss function was designed to solve some of the above-mentioned problems with smooth $L_{1}$ loss. Through normalization, a value between 0 and 1 can be obtained regardless of the value of the large coordinates. However, it is not trained on non-overlapping bounding boxes because the IoU values are 0 when there is no overlap. To secure these shortcomings, generalized IoU (GIoU) [43], distance IoU (DIoU) [22], and complete IoU (CIoU) have been proposed by adding a penalty term to the IoU loss function.

GIoU adds a penalty term that uses the smallest bounding box *E*, which covers both the predicted bounding box *B* and the ground truth bounding box $B^{gt}$, as in Equation (4). Its range is [−1, 1], which indicates that it does not have a normalized IoU form. Even when it does not overlap with the ground truth bounding box, it moves toward the ground truth bounding box in order to reduce the penalty term. However, when the predicted bounding box becomes large enough to include the ground truth bounding box, it operates in the same way as the IoU loss. It has also become even more sensitive to small offsets between small objects, which slows convergence and reduces performance [44]. DIoU compares the center coordinates of the bounding box and adds a distance-based penalty term. ρ(·) in Equation (5) is the Euclidean distance and *c* is the diagonal distance of the smallest bounding box, which contains *b* and $b^{gt}$. Compared to GIoU, convergence occurs faster in DIoU since distance-based horizontal and vertical directions are included.

(4) $$L_{GIoU}=1-IoU+\frac{\left|E\backslash \left(B\cup B^{gt}\right)\right|}{\left|E\right|}.$$

(5) $$L_{DIoU}=1-IoU+\frac{\rho^{2}\left(b,b^{gt}\right)}{c^{2}}.$$

To achieve successful bounding box regression, overlap areas, central point distance, and aspect ratio must all be taken into account. The CIoU loss adds a penalty term that is based on the aspect ratio to the DIoU loss, which reduces missing or false detection and improves the accuracy of bounding box detection. CIoU is defined as follows:

(6) $$L_{CIoU}=1-IoU+\frac{\rho^{2}\left(b,b^{gt}\right)}{c^{2}}+\alpha v,$$

where *v* measures the concordance of the aspect ratios and α regulates the balance between the non-overlapping cases and overlapping cases:

(7) $$v=\frac{4}{\pi^{2}}{\left(\arctan\frac{w^{gt}}{h^{gt}}-\arctan\frac{w}{h}\right)}^{2},$$

(8) $$\alpha =\frac{v}{(1-IoU)+v}.$$

However, CIoU loss still has problems with convergence speed and its performance is not ideal. Therefore, we proposed an idea to supplement the smooth $L_{1}$ loss and IoU-based loss problems by combining the loss functions. As a result, each loss function improved in thrombus detection performance due to complementary effects while training the network. Our new loss function, named SCIoU, was defined as follows:

(9) $$L_{SCIoU}=\delta smooth_{\ell_{1}}+\left(1-\delta \right)L_{CIoU},$$

where δ is a parameter that adjusts the weight of each loss function. The benefits of combining two different loss functions included the assistance of the smooth $L_{1}$ loss in the slow convergence, which is a disadvantage of CIoU, for quick convergence and the concentration on the regression analysis of the bounding box. The CIoU loss focuses on fine-tuning the predicted bounding box by considering the overlapping areas, center point distance, and aspect ratio.

Simultaneously, we replaced the cross-entropy loss, which is a mask loss, with a weighted binary focal loss to improve thrombus segmentation performance. It should be noted that mask loss was only calculated using positive sample ROIs.

Focal loss [45] applies a higher weight strategy for difficult examples and easily mis-classified cases, whereas low weight is used for easy examples. This idea is expressed mathematically in Equation (10), where *p* denotes the predicted probability of the ground truth class and $\alpha_{t}$ and γ are the hyper-parameters of the loss function:

(10) $$L_{FL}\left(p_{t}\right)=-\alpha_{t}{\left(1-p_{t}\right)}^{\gamma }\log\left(p_{t}\right).$$

In our function, modified focal loss, which we called weighted binary focal loss, was applied to binary classification. Instead of the $\alpha_{t}$ hyper-parameter of the traditional focal loss, the binary ground truth mask was multiplied to focus more on each of the positive and negative aspects. Finally, by adding positive and negative focal loss, weighting parameters were used to prevent the thrombus from being mis-segmented with an irregular shape that was obscured by the metal stent graft and improved segmentation performance. Our mask loss function was defined as follows:

(11) $$L_{WFL}\left(p_{t}\right)=\lambda L_{pos}\left(p_{t}\right)+L_{neg}\left(p_{t}\right),$$

(12) $$L_{pos}\left(p_{t}\right)=-\zeta_{t}{\left(1-p_{t}\right)}^{\gamma }\log\left(p_{t}\right),$$

(13) $$L_{neg}\left(p_{t}\right)=-\xi_{t}{\left(p_{t}\right)}^{\gamma }\log\left(1-p_{t}\right).$$

where λ denotes the parameter that controls the function by focusing more on the positive sample, ζ is a positive ground truth mask, and ξ is a negative ground truth mask.

## 4. Results

### 4.1. Dataset

In Table 1, we describe the overall characteristics of the dataset. Our dataset of AAA thrombus CTA scan images from 60 unique patients, which were generated from 2012 to 2020 at Gachon University Gil Medical Center in the Republic of Korea, contained the largest number of patients in a post-operation AAA thrombus dataset, to the best of our knowledge. Therefore, our dataset was well suited to establishing the robustness of the proposed method for thrombus variability. We were able to obtain CTA volume images using five different pieces of scanning equipment that were manufactured by the same Siemens company: SOMATOM Definition Edge, SOMATOM Definition Flash, SOMATOM Force, SOMATOM Emotion Duo, and Sensation 16.

The CTA volume images were converted from the Digital Imaging and Communications in Medicine (DICOM) file format into PNG-type two-dimensional images of 512 × 512 and consisted of data from the heart to below the pelvis. There were 49 to 206 slices for each patient and the axial slice interval had a value that ranged from 3 to 5 mm.

The basic characteristics of the study group were 46 male patients and 14 female patients. The male to female ratio was 3.28:1, which was similar to the statistics of the study in the United States (4:1) [46]. In general, AAAs occur more frequently in the elderly population (over 65 years) and in our dataset, male patients averaged 72 years of age and female patients averaged 78 years of age [47]. The proportion of images that had a ground truth label of thrombus out of the CTA slice images of all patients was 20%, with a standard deviation of 7%. Ground truth voxels were manually labeled with thrombus in the axial view direction by trained radiologists.

### 4.2. 3D Quantitative Metrics for Evaluation

The experimental results were evaluated using an evaluation index in two aspects: detection performance and segmentation performance. To evaluate and comprehensively verify the thrombus detection performance, we used the metrics of precision, recall, and F1 score. Precision represented the proportion of predicted positive cases that were accurately identified as ground truth positive and recall represented the proportion of ground truth positive cases that were accurately predicted as positive. True positive (TP) was defined as an IoU of 0.5 or higher. The F1 score is another general indicator of the precision and recall of the harmonic mean. It could be said that the greater the F1 score, the better the model detection performance. The precision, recall, and F1 score were calculated as follows:

(14) $$\mathrm{Precision}=\frac{\mathrm{TP}}{\mathrm{TP}+\mathrm{FP}},$$

(15) $$\mathrm{Recall}=\frac{\mathrm{TP}}{\mathrm{TP}+\mathrm{FN}},$$

(16) $$F1\;\mathrm{score}\;=\frac{2\times \;\mathrm{Precision}\;\times \;\mathrm{Recall}\;}{\;\mathrm{Precision}\;+\;\mathrm{Recall}\;}.$$

For segmentation results, region-based and distance-based measures are often used for segmentation evaluation. Therefore, we computed the total overlap (TO), Dice coefficient, Jaccard index, false negative rate (FN), and false positive rate (FP), as proposed in [48]. At the same voxel resolution, the segmented volume (source, S) that was obtained through our modified Mask R-CNN for each patient was restored to three dimensions and compared to the ground truth (target, T). The total overlap was calculated by dividing the intersection between two thrombus regions r in the S and T by the ground truth region in T and was expressed as follows:

(17) $$\mathrm{TO}=\frac{|S_{r}\cap T_{r}|}{|T_{r}|}.$$

The Dice coefficient was used to calculate the amount of spatial overlap between two thrombus regions. The Dice coefficient value is shown below:

(18) $$\mathrm{Dice}=2\frac{|S_{r}\cap T_{r}|}{|S_{r}\left|+\right|T_{r}|}.$$

The Jaccard index represented the area of overlap between the source thrombus region and the target thrombus region divided by the union region:

(19) $$\mathrm{Jaccard}=\frac{|S_{r}\cap T_{r}|}{|S_{r}\cup T_{r}|}.$$

A false negative (FN) was referred to as a type II error, which meant that the ground truth voxels failed to segment. A false positive (FP) was referred to as a type I error, which predicted voxels that were not ground truth voxels and was expressed as follows:

(20) $$\mathrm{FN}=\frac{|T_{r}-S_{r}|}{|T_{r}|},$$

(21) $$\mathrm{FP}=\frac{|S_{r}-T_{r}|}{|S_{r}|}.$$

### 4.3. Experiments

All experiments used a 4-fold cross-validation approach to decrease the possibility of biased testing and provide robustness to the results. We split our dataset into four different folds, then trained the model with three of the folds and tested the model with the remaining fold. We conducted four rounds, each of which used a different test fold, and calculated the average value of the performance metrics across the four rounds. We divided the dataset according to the patients, i.e., there were no overlapping patients in each fold.

We implemented the proposed method using Python and Pytorch library. The test environment was run on an Intel Core E5-2620 v4 CPU with a clock speed of 2.10 GHz and an NVIDIA TITAN RTX (24 GB RAM) graphics card. We employed the stochastic gradient descent (SGD) optimization method. We empirically set the initial learning rate to 5e-3 and the momentum to 9e-1. The code is available on GitHub (Link: https://github.com/AAA-improved-mask-rcnn).

### 4.4. Thrombus Detection Results

**Figure 3:**
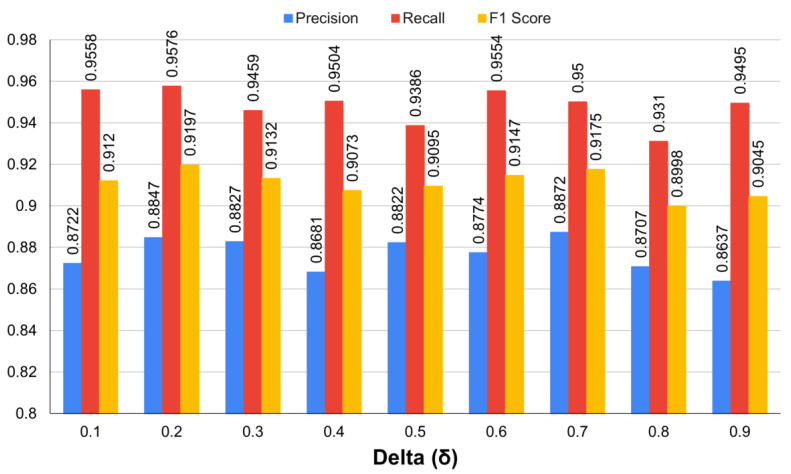
The evaluation results for different delta values of the SCIoU bounding box regression loss.

Before changing the segmentation loss function, we decided to set the bounding box loss function using the best regression performance. The hyper-parameter delta value that regulated the ratio was fixed to a value between 0.1 and 0.9 in order to find the optimal balance between the smooth L1 loss and the CIoU loss and then the performance evaluation was performed. Figure 3 displays the performance values for precision, recall, and F1 score for different delta values of the SCIoU loss function. When the delta value was 0.2, the precision value was 0.8847, the recall value was 0.9576, and the F1 score value was 0.9197, which were the highest values out of all of the evaluation indicators. Setting the delta value that was more focused on the smooth $L_{1}$ loss to 0.8 and 0.9 resulted in comparatively low values.

**Figure 4:**
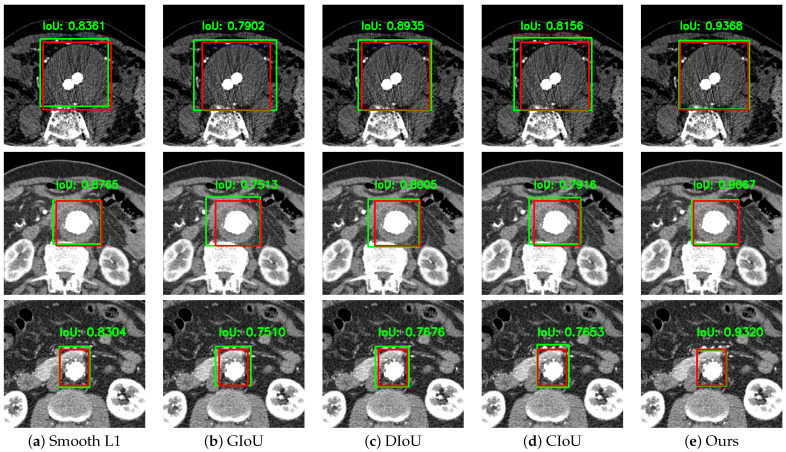
Detected thrombi: red boxes are ground truth voxels and green boxes are predicted voxels.

Our proposed SCIoU loss function had both the four-variable independent regression advantage of smooth $L_{1}$ loss and the center point distance and aspect ratio advantages of CIoU loss. Table 2 shows the results when the bounding box regression loss of Mask R-CNN was changed to the existing loss function that is widely used. Our SCIoU loss function had the highest values for F1 score and precision compared to the other loss functions and the recall value achieved 0.9576, which was 0.0005 lower than the vanilla Mask R-CNN that only uses the smooth $L_{1}$ loss. Figure 4 also shows that each coordinate was appropriately regressed while maintaining the ratio in contrast to IoU-based losses, which are more regressed than GT.

We compared the regression performance of the DeepAAA [35] and DetectNet [34] methods using our dataset. Table 3 shows that our proposed loss function outperformed the DeepAAA method using the smooth negative Dice coefficient and the DetectNet method using the $L_{1}$ loss in terms of recall. Our predicted bounding box regressed in a highly similar manner to the ground truth bounding box. Additionally, the evaluation showed that SCIoU had a higher precision value (0.8847) than the other models. Finally, the F1 score, which represents the harmony between precision and recall, also had a high value of 0.9197. It could be proved that the proposed loss function was efficient in the bounding box regression evaluation overall.

### 4.5. Thrombus Segmentation Results

Due to the similar intensities of the medical images to other tissues, thrombi can only be detected intermittently, even in images that do not contain thrombi. As a result, the segmentation performance was evaluated using an extraction algorithm for the detected thrombus from a medical diagnosis perspective. DeepAAA evaluated the image that was obtained when the largest diameter was greater than 3 cm by applying ellipse fitting. The vanilla Mask R-CNN and our proposed combined loss Mask R-CNN (CL Mask R-CNN) were both influenced by Lopez-Linares et al. [34] and used a continuous slice-based approach. It was confirmed that a thrombus had been found when the number of slice images of the detected thrombus was above a certain threshold in each patient. Since the patients in our dataset had at least eight thrombus images, segmentation was only conducted when more than eight consecutive thrombi were detected.

**Figure 5:**
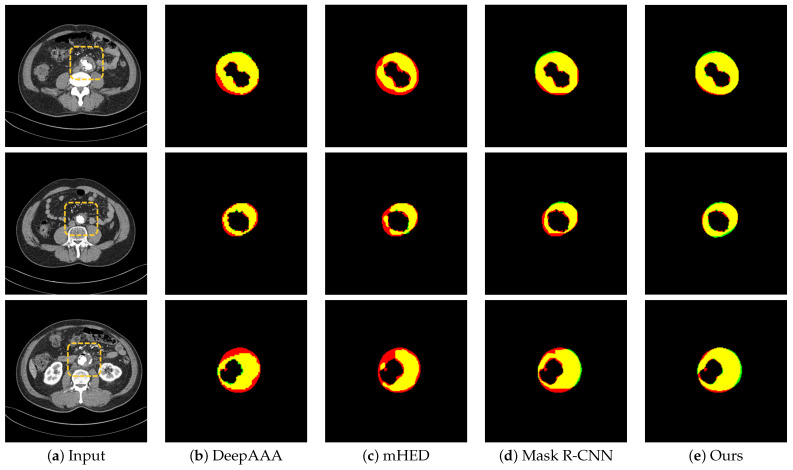
2D segmentation results of thrombi: (**a**) input image; (**b**–**e**) the segmentation results from each method. Yellow, red, and green regions represent true positive, false negative, and false positive results, respectively.

**Figure 6:**
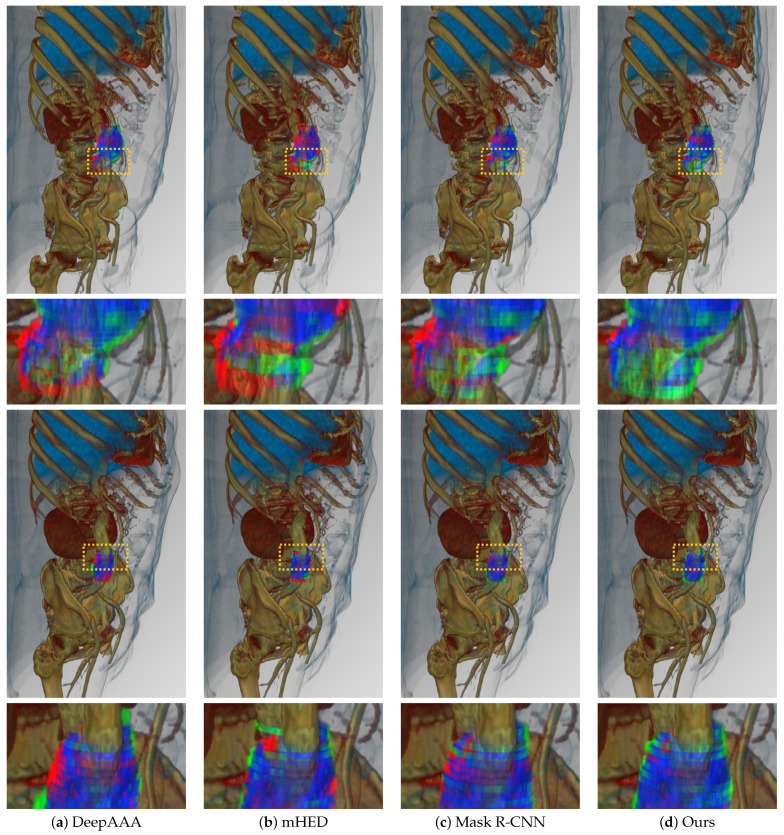
3D volume rendering results of segmentation after using the detection algorithm. The overlapping parts of the ground truth and prediction mask voxels from the model are represented in blue, false positives are in green, and false negatives are in red. Yellow box is represent the magnification region.

Table 4 shows the detailed thrombus segmentation experimental results for the five evaluation indicators. Compared to the two deep learning-based networks and the vanilla Mask R-CNN, our proposed CL Mask R-CNN achieved the highest segmentation performance of 0.8971 for the total overlap, 0.7163 for the Jaccard index, and 0.8267 for the Dice coefficient. In medical image segmentation, reducing false negatives is recommended over reducing false positives [49]. This is because false negatives can have severe consequences for patient health and false positives can be resolved later by trained radiologists. In this study, our method achieved the lowest false negative rate of 0.1029. Figure 5 shows the results of the visualization of the detected thrombi based on 2D images, which resulted in a significant reduction in false negatives. Furthermore, Figure 6 shows that false negatives decreased and false positives increased in the area where the qualitative evaluation was performed by converting the 2D results into 3D images.

## 5. Conclusions and Future Work

Previous research has concentrated on thrombus segmentation rather than thrombus detection and Claridge et al. reported that only 65% of thrombi were detected by trained radiologists [17]. As such, detection is as crucial as segmentation and because it is related to patient mortality, there should be no missed or incorrect detections. Therefore, we proposed to change the loss function of the Mask R-CNN for thrombus detection and the segmentation of 2D-based images that were obtained from the CTA dataset. The proposed method was able to detect thrombi correctly while also precisely segmenting thrombi in each CTA image slice. As can be seen in Table 2 and Table 3, our method achieved a high detection performance. The segmentation results also outperformed the existing deep learning methods and the false negative rate was significantly reduced. Furthermore, our method has significant clinical value because it could reduce the possibility of thrombi going undetected and support radiologists in making clinical decisions for AAA patients.

The model that was adopted in this paper is a Mask R-CNN, which is based on instance segmentation. Recently, various new methods have been developed, such as a Mask Scoring R-CNN, which can improve the performance of Mask R-CNN. In future work, we plan to use these models as backbone networks to achieve high accuracy for thrombus detection and segmentation.

## Figures and Tables

**Table 1:** Detailed description of the characteristics of our dataset.

Characteristics	Detailed Information
Number of patients	60 (Unique)
Number of CTA slice images	8739
Dates of captured images	2012–2020
Number of pieces of equipment	5
Image size	512 × 512
Gender proportion	76% male; 24% female
Mean age	72 years (Male); 78 years (Female)
Thrombus proportion in all slice images	20%;7%(SD)

**Table 2:** The results of the bounding box regression evaluation of basic regression loss functions and our proposed SCIoU regression loss function.

Network	Precision	Recall	F1 Score
Smooth L1 [21]	0.8694	0.9581	0.9115
GIoU [43]	0.8409	0.9491	0.8917
DIoU [22]	0.8273	0.9701	0.893
CIoU [22]	0.8553	0.9455	0.8981
SCIoU (Ours)	0.8847	0.9576	0.9197

**Table 3:** The results of comparing various thrombus detection models when the bounding box regression was changed from the vanilla Mask R-CNN to the proposed SCIoU function.

Network	Precision	Recall	F1 Score
DeepAAA [35]	0.8813	0.9103	0.8955
DetectNet [34]	0.8321	0.9020	0.8656
Mask R-CNN [38]	0.8694	0.9581	0.9115
Mask R-CNN (Ours)	0.8847	0.9576	0.9197

**Table 4:** The results of thrombus segmentation when applying a detection algorithm for medical relevance. Our proposed CL Mask R-CNN was changed in both the bounding box regression loss function and the segmentation mask loss function.

Network	Overlap	Jaccard	Dice	FN	FP
DeepAAA [35]	0.7470	0.6808	0.7923	0.2530	0.1394
mHED [34]	0.7453	0.6676	0.7864	0.2546	0.1284
Mask R-CNN [38]	0.8615	0.7085	0.8181	0.1385	0.1946
CL Mask R-CNN	0.8971	0.7163	0.8267	0.1029	0.2095

## Data Availability

The data are not publicly available due to privacy restrictions.

## References

[1] Rissland P., Alemu Y., Einav S., Ricotta J., Bluestein D. (2009). Abdominal aortic aneurysm risk of rupture: Patient-specific FSI simulations using anisotropic model. J. Biomech. Eng..

[2] Lederle F.A. (2009). Abdominal aortic aneurysm. Ann. Intern. Med..

[3] Wilmink A., Hubbard C.S., Day N., Quick C. (2001). The incidence of small abdominal aortic aneurysms and the change in normal infrarenal aortic diameter: Implications for screening. Eur. J. Vasc. Endovasc. Surg..

[4] Lederle F.A., Johnson G.R., Wilson S.E., Chute E.P., Hye R.J., Makaroun M.S., Barone G.W., Bandyk D., Moneta G.L., Makhoul R.G. (2000). The aneurysm detection and management study screening program: Validation cohort and final results. Arch. Intern. Med..

[5] Nordon I.M., Hinchliffe R.J., Loftus I.M., Thompson M.M. (2011). Pathophysiology and epidemiology of abdominal aortic aneurysms. Nat. Rev. Cardiol..

[6] Acosta S., Ögren M., Bengtsson H., Bergqvist D., Lindblad B., Zdanowski Z. (2006). Increasing incidence of ruptured abdominal aortic aneurysm: A population-based study. J. Vasc. Surg..

[7] Chaikof E.L., Dalman R.L., Eskandari M.K., Jackson B.M., Lee W.A., Mansour M.A., Mastracci T.M., Mell M., Murad M.H., Nguyen L.L. (2018). The Society for Vascular Surgery practice guidelines on the care of patients with an abdominal aortic aneurysm. J. Vasc. Surg..

[8] Fillinger M.F. (1999). Postoperative imaging after endovascular AAA repair. Seminars in Vascular Surgery.

[9] Maiora J., Ayerdi B., Graña M. (2014). Random forest active learning for AAA thrombus segmentation in computed tomography angiography images. Neurocomputing.

[10] Moxon J.V., Parr A., Emeto T.I., Walker P., Norman P.E., Golledge J. (2010). Diagnosis and monitoring of abdominal aortic aneurysm: Current status and future prospects. Curr. Probl. Cardiol..

[11] Duquette A.A., Jodoin P.M., Bouchot O., Lalande A. (2012). 3D segmentation of abdominal aorta from CT-scan and MR images. Comput. Med. Imaging Graph..

[12] Freiman M., Esses S.J., Joskowicz L., Sosna J. (2010). An iterative model-constrained graph-cut algorithm for abdominal aortic aneurysm thrombus segmentation. Proceedings of the 2010 IEEE International Symposium on Biomedical Imaging: From Nano to Macro.

[13] Demirci S., Lejeune G., Navab N. (2009). Hybrid deformable model for aneurysm segmentation. Proceedings of the 2009 IEEE International Symposium on Biomedical Imaging: From Nano to Macro.

[14] Lalys F., Yan V., Kaladji A., Lucas A., Esneault S. (2017). Generic thrombus segmentation from pre-and post-operative CTA. Int. J. Comput. Assist. Radiol. Surg..

[15] Siriapisith T., Kusakunniran W., Haddawy P. (2018). Outer wall segmentation of abdominal aortic aneurysm by variable neighborhood search through intensity and gradient spaces. J. Digit. Imaging.

[16] Zohios C., Kossioris G., Papaharilaou Y. (2012). Geometrical methods for level set based abdominal aortic aneurysm thrombus and outer wall 2D image segmentation. Comput. Methods Programs Biomed..

[17] Claridge R., Arnold S., Morrison N., van Rij A.M. (2017). Measuring abdominal aortic diameters in routine abdominal computed tomography scans and implications for abdominal aortic aneurysm screening. J. Vasc. Surg..

[18] Krizhevsky A., Sutskever I., Hinton G.E. (2012). Imagenet classification with deep convolutional neural networks. Adv. Neural Inf. Process. Syst..

[19] Chen L.C., Papandreou G., Kokkinos I., Murphy K., Yuille A.L. (2017). Deeplab: Semantic image segmentation with deep convolutional nets, atrous convolution, and fully connected crfs. IEEE Trans. Pattern Anal. Mach. Intell..

[20] Minaee S., Boykov Y.Y., Porikli F., Plaza A.J., Kehtarnavaz N., Terzopoulos D. (2021). Image segmentation using deep learning: A survey. IEEE Trans. Pattern Anal. Mach. Intell..

[21] Girshick R. Fast r-cnn. Proceedings of the IEEE International Conference on Computer Vision.

[22] Zheng Z., Wang P., Liu W., Li J., Ye R., Ren D. Distance-IoU loss: Faster and better learning for bounding box regression. Proceedings of the AAAI Conference on Artificial Intelligence.

[23] Babin D., Pižurica A., Velicki L., Matić V., Galić I., Leventić H., Zlokolica V., Philips W. (2018). Skeletonization method for vessel delineation of arteriovenous malformation. Comput. Biol. Med..

[24] Babin D., Vansteenkiste E., Pižurica A., Philips W. (2012). Centerline calculation for extracting abdominal aorta in 3-D MRI images. Proceedings of the 2012 Annual International Conference of the IEEE Engineering in Medicine and Biology Society.

[25] Babin D., Devos D., Pižurica A., Westenberg J., Vansteenkiste E., Philips W. (2014). Robust segmentation methods with an application to aortic pulse wave velocity calculation. Comput. Med. Imaging Graph..

[26] Babin D., Devos D., Platiša L., Jovanov L., Habijan M., Leventić H., Philips W. (2020). Segmentation of phase-contrast mr images for aortic pulse wave velocity measurements. Proceedings of the International Conference on Advanced Concepts for Intelligent Vision Systems.

[27] Babin D., Vansteenkiste E., Pizurica A., Philips W. (2009). Segmentation and length measurement of the abdominal blood vessels in 3-D MRI images. Proceedings of the 2009 Annual International Conference of the IEEE Engineering in Medicine and Biology Society.

[28] de Bruijne M., van Ginneken B., Viergever M.A., Niessen W.J. (2004). Interactive segmentation of abdominal aortic aneurysms in CTA images. Med. Image Anal..

[29] Macía I., Legarreta J.H., Paloc C., Graña M., Maiora J., García G., Blas M.D. (2009). Segmentation of abdominal aortic aneurysms in CT images using a radial model approach. Proceedings of the International Conference on Intelligent Data Engineering and Automated Learning.

[30] Joldes G.R., Miller K., Wittek A., Forsythe R.O., Newby D.E., Doyle B.J. (2017). BioPARR: A software system for estimating the rupture potential index for abdominal aortic aneurysms. Sci. Rep..

[31] Zheng J.Q., Zhou X.Y., Li Q.B., Riga C., Yang G.Z. (2018). Abdominal aortic aneurysm segmentation with a small number of training subjects. arXiv.

[32] Hong H.A., Sheikh U. (2016). Automatic detection, segmentation and classification of abdominal aortic aneurysm using deep learning. Proceedings of the 2016 IEEE 12th International Colloquium on Signal Processing & Its Applications (CSPA).

[33] Wang D., Zhang R., Zhu J., Teng Z., Huang Y., Spiga F., Du M.H.F., Gillard J.H., Lu Q., Liò P. Neural network fusion: A novel CT-MR aortic aneurysm image segmentation method. Proceedings of the Medical Imaging 2018: Image Processing; International Society for Optics and Photonics.

[34] López-Linares K., Aranjuelo N., Kabongo L., Maclair G., Lete N., Ceresa M., García-Familiar A., Macía I., Ballester M.A.G. (2018). Fully automatic detection and segmentation of abdominal aortic thrombus in post-operative CTA images using deep convolutional neural networks. Med. Image Anal..

[35] Lu J.T., Brooks R., Hahn S., Chen J., Buch V., Kotecha G., Andriole K.P., Ghoshhajra B., Pinto J., Vozila P. (2019). DeepAAA: Clinically applicable and generalizable detection of abdominal aortic aneurysm using deep learning. Proceedings of the International Conference on Medical Image Computing and Computer-Assisted Intervention.

[36] Nanni L., Cuza D., Lumini A., Loreggia A., Brahnam S. (2021). Deep ensembles in bioimage segmentation. arXiv.

[37] Dong B., Wang W., Fan D.P., Li J., Fu H., Shao L. (2021). Polyp-PVT: Polyp Segmentation with Pyramid Vision Transformers. arXiv.

[38] He K., Gkioxari G., Dollár P., Girshick R. Mask r-cnn. Proceedings of the IEEE International Conference on Computer Vision.

[39] Lin T.Y., Dollár P., Girshick R., He K., Hariharan B., Belongie S. Feature pyramid networks for object detection. Proceedings of the IEEE Conference on Computer Vision and Pattern Recognition.

[40] He K., Zhang X., Ren S., Sun J. Deep residual learning for image recognition. Proceedings of the IEEE Conference on Computer Vision and Pattern Recognition.

[41] Long J., Shelhamer E., Darrell T. Fully convolutional networks for semantic segmentation. Proceedings of the IEEE Conference on Computer Vision and Pattern Recognition.

[42] Ren S., He K., Girshick R., Sun J. (2016). Faster R-CNN: Towards real-time object detection with region proposal networks. IEEE Trans. Pattern Anal. Mach. Intell..

[43] Rezatofighi H., Tsoi N., Gwak J., Sadeghian A., Reid I., Savarese S. Generalized intersection over union: A metric and a loss for bounding box regression. Proceedings of the IEEE/CVF Conference on Computer Vision and Pattern Recognition.

[44] Xu C., Wang J., Yang W., Yu L. Dot Distance for Tiny Object Detection in Aerial Images. Proceedings of the IEEE/CVF Conference on Computer Vision and Pattern Recognition.

[45] Lin T.Y., Goyal P., Girshick R., He K., Dollár P. Focal loss for dense object detection. Proceedings of the IEEE International Conference on Computer Vision.

[46] Harthun N.L. (2008). Current issues in the treatment of women with abdominal aortic aneurysm. Gend. Med..

[47] Vardulaki K., Walker N., Day N., Duffy S., Ashton H., Scott R. (2000). Quantifying the risks of hypertension, age, sex and smoking in patients with abdominal aortic aneurysm. J. Br. Surg..

[48] Tustison N.J., Gee J.C. (2009). Introducing Dice, Jaccard, and other label overlap measures to ITK. Insight J..

[49] Burt T., Button K., Thom H., Noveck R., Munafò M.R. (2017). The Burden of the “False-Negatives” in Clinical Development: Analyses of Current and Alternative Scenarios and Corrective Measures. Clin. Transl. Sci..

